# Exploring the effects of high temperature on mortality in four cities in the Philippines using various heat wave definitions in different mortality subgroups

**DOI:** 10.1080/16549716.2017.1368969

**Published:** 2017-09-15

**Authors:** Xerxes T. Seposo, Tran Ngoc Dang, Yasushi Honda

**Affiliations:** ^a^ Graduate School of Comprehensive Human Sciences, University of Tsukuba, Tsukuba City, Japan; ^b^ Department of Environmental Health, Faculty of Public Health, University of Medicine and Pharmacy, Ho Chi Minh City, Vietnam; ^c^ Faculty of Health and Sports Sciences, University of Tsukuba, Tsukuba City, Japan

**Keywords:** Heat wave, Philippines, DLNM, mortality sub-groups, tropical

## Abstract

**Background**: Sustained high temperatures, specifically heat waves (HW), increase the risk of dying, especially among risk populations, which are highly vulnerable to its additional effect. In developing countries, there are only a few studies which focused on the magnitude of the risks attributed to HWs.

**Objectives**: This study explored the HW effects using 15 HW definitions through the combination of duration (> 2, > 4, and > 7 consecutive days) and intensity (at the ≥ 90th, ≥ 95th, ≥ 97th, ≥ 98th, and ≥ 99th temperature percentiles).

**Methods**: Daily mortality count data from 2006–2010 were obtained from the four tropical cities of the Philippines, and were further stratified by mortality sub-groups, such as cause of death, sex, and age. The same period of daily maximum temperature and relative humidity were also collected. We used a distributed lag non-linear model to determine the risks associated with the main temperature effects, as well as the added HW effects.

**Results**: It has been observed that the main temperature effects comprise a substantial portion of the risks compared to the HW effects, even across the mortality sub-groups. Further stratification by the sub-groups showed significant HW effects among the young and male populations.

**Conclusions**: Results of this study can be of use to improve (1) candidate HW definition identification/selection, and (2) risk population-specific strategies, taking into consideration the risk attributions.

## Background

Globally, numerous studies have reported that the temperature-mortality relationship tends to follow U-, V-, and J-shaped non-linear patterns, whereby risks in the extreme ends constitute higher risks relative to the minimum mortality temperature (MMT) [1–5]. These extreme temperatures account for greater risks, and to a certain extent, when analyzed separately, in relation to duration and intensity, which are the length of day and threshold temperature, respectively, constitute the notion of heat waves (HW) [6–10]. However, there is yet to be a unifying notion of standardizing HW definitions as these vary with respect to duration and intensity of temperature in a specific geographical area [11,12]. Nevertheless, various studies have similar observations of increasing risks with increasing duration and intensity, particularly on HW days [9–11,13,14]. Major heat wave events have been recorded in the past years, such as the 1995 Chicago heat wave event, which claimed more than 600 excess deaths [15] and, in the summer of 2003, more than 70,000 excess deaths were recorded in Europe, which was partially attributed to the HW days [16].

Extreme temperature events have posed greater risks to health across the globe, and, according to McMichael *et al*. [4], adverse effects of these extreme temperatures will manifest mostly in cities of low- and middle-income countries, regardless of climatological orientation, whether in temperate, sub-tropical, or tropical context. Research has been done in temperate countries with regard to HW effects, with similar patterns of increased risk in increasing duration and intensity [9,10,13,14,17,18]. Most studies in sub-tropical and tropical cities have observed increased risks at both sides of the temperature extremes, with less or no emphasis on HW [4,19–22]. The Philippines, a middle-income country, has not yet fully integrated heat-health action plans, which include the HW risk estimation, regardless of the fact that observed relative risks (RR) are evident in the extreme high temperature [20]. In order ascertain the extent of HW effects, we classified the temperature effects into two; namely, main and added effects, whereby the main effects constitute the independent effect of daily temperature, while the added effects are due to the duration of sustained heat on consecutive days, otherwise known as the HW effect, and consequently estimated the respective associated risks [9,23].

This study builds upon the existing knowledge of temperature-mortality risks with the emphasis on HW in tropical cities, in order to determine the degree or extent of risk attributed to the main and added effects. We believe that the determination of these risks will be instrumental for policymakers with respect to crafting climate-driven and health-oriented plans for both the general and risk populations.

## Methods

### Descriptive summary of study sites and geographical locations

**Figure 1.**
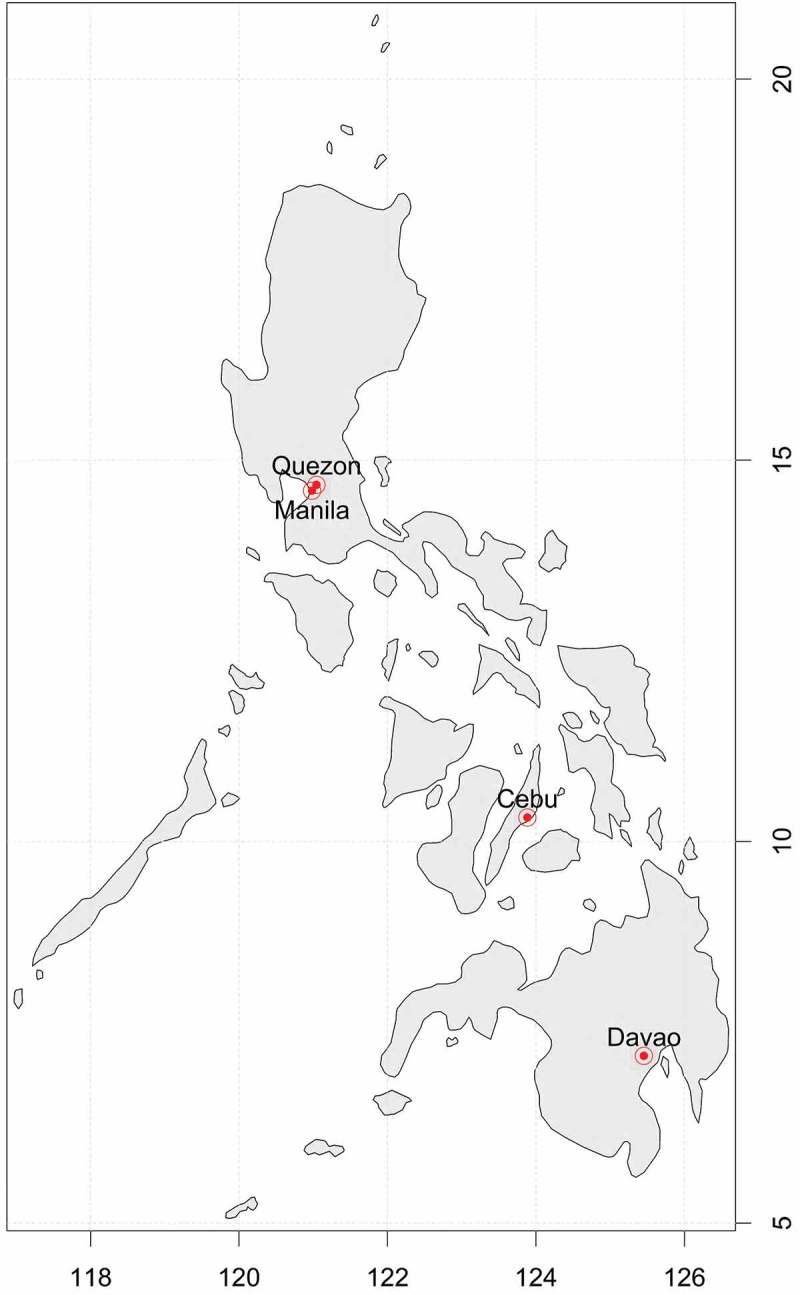
Geographic location of the four tropical cities.

The study sites are located in the four major cities of the Philippines, namely Manila, Quezon, Cebu, and Davao, which comprised 7% of the country’s population in 2010 [27]. Table 1 shows the demographic characteristics of the specific study sites. Manila and Quezon cities are situated in the north of the country, while Cebu and Davao are located in the central and southern regions, respectively; Figure 1. The Philippine climate types were initially classified in the 1920s by Rev. José Coronas. S.J., the Chief of the Manila Observatory’s Meteorological Division. In the succeeding years, the Philippine Atmospheric Geophysical and Astronomical Services Administration (PAGASA) adopted and modified the classification, which is now known as the Modified Coronas classification [26]. There are four climate types, namely Type I, II, III, and IV. Type I has an almost completely dry season from November to April, with a pronounced wet season from May to October, while Type II has a seasonal peak of rainfall from November to December. Type III, on the other hand, resembles Type I, but with maximum rainfall periods from May to October. Lastly, Type IV has a more or less even distribution of rainfall year-round [26,28].

**Table 1.** Demographic summary of the study sites.

City	Coordinates*^a^*	Land area*^b^* (in sq. km.)	Population*^b^*	Climate type*^c^*
Manila	14.5995 ⁰N, 120.9842 ⁰E	39	1,652,171	Type I
Quezon	14.6760 ⁰N, 121.0437 ⁰E	165	2,761,720	Type I
Cebu	10.3157 ⁰N, 123.8854 ⁰E	315	866,171	Type III
Davao	7.1907 ⁰N, 125.4553 ⁰E	2,444	1,449,296	Type IV

*^a^*Longitude and latitude coordinates [24].

*^b^*2010 Population Census [25].

*^c^*Based on the Modified Coronas classification [26].

### Meteorological and mortality data

We acquired the daily 2006–2010 meteorological data from the Philippine Atmospheric Geophysical and Astronomical Services Administration (PAGASA), with the same period of mortality data from the Philippine Statistics Authority – National Statistics Office (PSA-NSO). We further classified mortality into different risk groups, such as cause of death, sex, and age. Cause of death was coded by using the International Classification of Diseases (ICD)-10, with I00–I99 for cardiovascular-related mortality, and J00–J99 for respiratory-related mortality, whereas age was stratified into three groups, namely, 0–14 years old, 15–64 years old, and > 64 years old (in Table 2).

**Table 2.** Descriptive statistics of the population-adjusted mortality and meteorological variables of the four tropical cities in the Philippines.

Variables	Manila City	Cebu City	Davao City	Quezon City
*All-cause mortality^a^*^,^*^b^*	3.07	2.71	1.66	1.80
*Cause-specific mortality*				
Cardiovascular	0.90	0.84	0.59	0.63
Respiratory	0.38	0.29	0.14	0.21
*Age-specific mortality*				
0–14 years old	0.48	0.31	0.13	0.22
15–64 years old	1.58	1.37	0.87	0.89
> 64 years old	1.01	1.04	0.66	0.69
*Sex-specific mortality*				
Female	1.33	1.17	0.66	0.76
Male	1.75	1.56	1.00	1.04
*Meteorological variables* (mean ± SD)				
*Dry season maximum temperature*	31.7 ± 2.12	31.1 ± 1.73	31.9 ± 1.85	32.4 ± 2.31
*Average relative humidity*	70.8 ± 7.50	84.4 ± 5.90	81.4 ± 5.40	74.9 ± 8.41

*^a^*population-adjusted rates using the 2010 population census.

*^b^*per 100,000 population.

### Statistical analyses

In examining the relative risks (RR) with respect to the lag and temperature dimensions, we used Quasi-Poisson regression, to account for over-dispersion coupled with distributed lag non-linear model (DLNM), as seen in Equation (1).

(1)
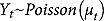

(2)
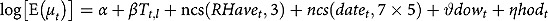

*log*[E(*μ_t_*)] is the expected daily mortality count on day *t; α* is the intercept; *T_t,l_* is the matrix from the cross-basis function of DLNM applied on temperature, *T*, on lag days, *l; β* is the vector of coefficient of the matrix; *ncs* is a spline function; *RHave_t_* is the average relative humidity with 3 df; *date_t_* controls for the seasonal trends with 7 df/year for 5 years; *dow_t_* is the day of the week with vector of coefficient 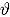; and *hod_t_* is a binary variable for public holidays with 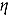 vector of coefficient.

In measuring the added effect of HW, we introduced a binary HW variable into Equation (1), with a value of 0 for non-HW day, otherwise 1 for HW day. We further restricted the analysis to the dry season, to avoid extreme low temperature effects in the analysis. After the determination of the city-specific risk curves in the first stage analysis, we pooled the estimated risk curves across the cities and generated a pooled risk pattern via a random effects meta-analysis with similar methodological specifications specified in the methodological paper by Gasparrini and Armstrong [9].

### HW definitions

In this study, since there is no standard definition for heat waves, we used the 2-, 4-, and 7-day duration with different intensities (90th, 95th, 97th, 98th, and 99th) as candidate HW definitions, which were also based on previous studies [9–11,13,18,23]. The selected intensities were city-specific, restricted to the dry season, which starts from December and ends in May. HW indicator counted as 1 if the day exceeds the temperature threshold in a given duration, otherwise, 0. HW effects were also estimated using the same meta-analytic techniques in the main temperature effects. Percent change [(RR-1) × 100] was calculated in reference to the 75th temperature percentile.

All statistical analyses were done using R Statistical Programming.

## Results

Among the four cities, most of the mortality counts were recorded in Manila City, with substantial proportions of mortality from Quezon, Davao, and Cebu cities. Between the cause-specific mortality sub-groups, cardiovascular cases were more prevalent, which were twice the number of recorded respiratory cases. Although most of the mortality was observed in the 15–64 year group when stratified by age, there was not much difference with the male and female mortality distributions. On the other hand, meteorological variables in the dry season, such as temperature and relative humidity, were relatively the same across different cities.

As seen in Table 3, the varying duration and intensity affected the detection of the HW days. Most HW days were recorded in Manila for HW days beyond the 90th temperature percentile, and continuing for more than 2 days. We can observe that, as the duration increases, and as the intensity increases, the number of HW days being detected becomes less.

**Table 3.** Summary statistics of heat wave days based on different duration (> 2, > 4, > 7 days) and intensity (90th, 95th, 97th, 98th, 99th) and the total number of days from December to May (of 2006–2010).

	Heat wave days															
	2 days					4 days					7 days					
City	90^th^	95^th^	97^th^	98^th^	99^th^	90^th^	95^th^	97^th^	98^th^	99^th^	90^th^	95^th^	97^th^	98^th^	99^th^	Total number of days
Manila	57	35	19	10	4	39	26	13	7	2	29	20	7	4	0	911
Cebu	44	44	6	4	2	13	13	0	0	0	1	1	0	0	0	911
Davao	50	37	12	6	5	24	17	3	0	0	15	12	0	0	0	911
Quezon	52	31	12	8	1	26	16	6	4	0	16	11	3	1	0	911

Table 4 shows the percentage change due to either the main temperature or HW effects. It can be observed that main temperature accounts for larger risks compared to HW days, in reference to the 75th temperature percentile, except for the 4-day, 99th temperature percentile. This may have been caused by the small number of HW days detected. Furthermore, there were no HW days observed at the 7-day, 99th temperature percentile.

**Table 4.** Percentage change attributed to main temperature effects and added HW effects in different duration and intensity among all-cause mortality in reference to the 75th temperature percentile.

Duration	Intensity	% Change Main temperature effects	95% CI	% Change HW effects	95% CI
2 days	90^th^	12.5	(4.70–20.9)	−2.50	(−6.40–1.70)
	95^th^	14.1	(4.00–25.2)	−1.30	(−5.80–3.40)
	97^th^	22.8	(5.20–43.4)	−5.40	(−11.6–1.20)
	98^th^	17.1	(−5.20–44.6)	−4.60	(−11.9–3.40)
	99^th^	16.2	(−9.40–49.0)	4.10	(−11.1–22.1)
4 days	90^th^	13.5	(3.10–25.1)	−0.20	(−10.3–11.0)
	95^th^	13.7	(2.50–26.2)	−0.30	(−6.00–5.80)
	97^th^	26.2	(9.10 – 46.0)	−0.90	(−9.80–8.80)
	98^th^	15.3	(−3.60–37.8)	2.20	(−10.1–16.3)
	99^th^	4.40	(−12.8–24.9)	29.3	(−4.40–74.8)
7 days	90^th^	11.2	(0.70–22.8)	3.40	(−7.20–15.1)
	95^th^	11.8	(−0.30–25.4)	2.60	(−4.80–10.7)
	97^th^	16.0	(7.80–24.7)	−0.90	(−13.8–13.9)
	98^th^	18.2	(8.50–28.7)	3.90	(−14.0–25.6)

Risks were higher in main temperature effects compared to the HW effects, with most of the HW effects having negative change. Either of the cardiovascular- or respiratory-related causes of mortality exhibited significant negative changes in the HW effects, while, in the sex-specific analysis, males have higher risks during HW days compared to females. Although risk estimates in the elderly were predominantly higher compared to the other age groups, these effects were statistically insignificant. On the other hand, the younger population (0–14 years old) were observed to be more susceptible to HW days, with significant effects at the 2-day, 90th and 95th temperature percentiles, and the 4-day, 95th temperature percentile, respectively.

## Discussions

Risks associated with main temperature effects were found to be greater than those of the HW effects; except for the 4-day, 99th temperature percentile definition. Although HW effects were statistically insignificant among all-cause mortality, we found statistically significant relationships in the stratified mortality sub-groups, particularly for the 0–14 years old and the male population. Results of this study can be used for policy-relevant strategies unique for the risk populations, taking into consideration the magnitude of the HW risks, in relation to the HW definitions.

In Table 3, although there might be intense HW events, which may last for a week or more, these events were rare; exemplified by 7-day duration with 99th temperature percentile yielding zero HW days. With all-cause mortality, we have not observed any definite risk patterns in relation to the increase or decrease of risk among different HW definitions; however, risks associated with the main effects were evidently greater than the HW effects, except for the 4-day, 99th temperature percentile definition (in Table 4), which may be due to the low number of HW days detected.

Even though the HW effects were statistically insignificant under all-cause mortality, significant effects estimates were observed when stratified by different sub-groups, particularly among 0–14 year olds and male risk groups. Temperature affects the thermoregulatory capacity of the person, which can be detrimental to overall health [14]. Occasional extreme heat days, such as HW, may pose even greater risks to the body, which is already at the current state of risk from the main temperature [10], especially for the at risk populations. Other studies have observed higher risks among the elderly due to the decreased thermoregulatory capacity when a person ages [20,29]. Factors such as dehydration affect the elderly through decreased sweat production and reduced direct loss of skin warmth, thereby decreasing the thermoregulation and thermal homeostasis [30,31]. Although the elderly people have greater effects estimates compared to the other age-specific risk groups, as seen in Table 5, the estimates were not significant. On the other hand, significant increased risks during HW days were observed in the 0–14 years old age group. Children are vulnerable to heat stress as their bodies are less adaptable to heat compared to adults [32]. Also, sweating capacity is lower in children than in adults, which results in a reduced dissipation of heat. The heightened heat exposure when playing outdoors further increases the risks, since the children do not instinctively replace their fluids or limit their exercise, even during extreme heat episodes [33].

**Table 5.** Percentage change of the main and HW effects by different risk populations in different duration and intensities with reference to the 75th temperature percentile.

Mortality sub-group	Duration	Intensity	% Change Main temperature effects	95% CI	% Change HW effects	95% CI
Cardiovascular	2 days	90^th^	17.9	(6.40–30.7)	−2.6	(−9.10–4.50)
		95^th^	19.1	(3.80–36.6)	−2	(−9.60–6.30)
		97^th^	25.3	(2.70–52.9)**	−3.1	(−16.6–12.7)
		98^th^	17	(−16.2–63.3)***	−7.7	(−19.7–6.00)
		99^th^	14.3	(−20.8 – 65.0)	8.5	(−14.0–36.8)
	4 days	90^th^	18.6	(5.90–32.9)	2.3	(−6.40–11.8)
		95^th^	21.5	(7.00–37.9)	−1.7	(−11.3–9.00)
		97^th^	21.5	(1.50–45.3)	9	(−10.6–32.9)
		98^th^	10.7	(−28.5–71.4)**	5.1	(−15.0–30.0)
		99^th^	−14.4	(−37.0–16.3)	26.7	(−23.8–111)
	7 days	90^th^	15.3	(2.30–30.1)	2.5	(−8.50–14.8)
		95^th^	12	(−3.20–29.6)*	10.4	(−3.30–25.9)
		97^th^	18	(4.20–33.5)	−2.5	(−37.9–53.1)**
		98^th^	16.3	(−15.1–59.2)*	−10	(−35.2–25.0)
Respiratory	2 days	90^th^	31.5	(14.1–51.5)	−9.6	(−19.4–1.40)
		95^th^	31.3	(5.20– 64.0)*	−9.5	(−31.1–18.8) **
		97^th^	56.3	(10.4–121)*	−18.7	(−36.2–3.50)
		98^th^	37.4	(−18.8–132)**	−3.1	(−22.0–20.2)
		99^th^	42.5	(−17.7–147)**	−2.5	(−28.4–32.8)
	4 days	90^th^	37.3	(16.2–62.3)	−11.9	(−24.1–2.20)
		95^th^	30.5	(1.00–68.7)	−6.5	(−28.8–22.9) *
		97^th^	34	(−29.5–155)***	5	(−23.6–44.4)
		98^th^	−10.6	(−63.4–119)**	31.2	(−6.10–83.2)
		99^th^	−15.7	(−52.3–48.8)	−54.8	(−90.4–112)
	7 days	90^th^	23.2	(6.30–42.8)	4.5	(−15.5–29.3)
		95^th^	27.1	(6.40–51.8)	6.6	(−12.4–29.7)
		97^th^	10.3	(−19.8–51.8)	5.1	(−26.7–50.7)
		98^th^	8.2	(−35.4–81.2)*	2.5	(−41.1–78.4)
Female	2 days	90^th^	16.7	(4.70–30.1) *	−5.4	(−11.1–0.60)
		95^th^	21.8	(5.20–40.9) **	−4.6	(−11.0–2.30)
		97^th^	30.2	(15.8–46.5)	−3.4	(−12.9–7.10)
		98^th^	28.5	(12.6–46.5)	−3.6	(−17.1–12.2)
		99^th^	24	(2.20–50.4)	5	(−12.0–25.4)
	4 days	90^th^	20.3	(7.40–34.7)	−3.8	(−11.1–4.10)
		95^th^	20.7	(7.30–35.7)	−2.8	(−11.3–6.60)
		97^th^	30.8	(13.1–51.3)	0.7	(−12.5–15.9)
		98^th^	27.1	(7.70–50.0)	1.7	(−16.1–23.3)
		99^th^	24.4	(−3.60–60.5)	18	(−23.4–81.7)
	7 days	90^th^	18.5	(3.70–35.5)**	−3	(−12.6–7.70)
		95^th^	18.7	(2.30–37.7)**	−1	(−11.9–11.2)
		97^th^	22.7	(5.20–43.1)	−3.2	(−20.3–17.6)
		98^th^	27.1	(11.8–44.6)	−9.8	(−32.5–20.6)
Male	2 days	90^th^	10	(3.90–16.4)	−0.1	(−6.60–6.90)
		95^th^	9.8	(2.80–17.3)	1.9	(−7.60–12.4)*
		97^th^	21.4	(5.70–39.3)	−7.1	(−15.5–2.30)
		98^th^	13.4	(−11.9–46.0)**	−4.7	(−16.4–8.70)
		99^th^	12.3	(−14.8–47.9)***	2.8	(−15.9–25.6)
	4 days	90^th^	12	(4.70–19.8)	2.1	(−9.50–15.1)**
		95^th^	11.4	(3.10–20.4)	2	(−5.40–10.1)
		97^th^	20.8	(0.30–45.6)*	−2.3	(−13.4–10.2)
		98^th^	6.8	(−18.1–39.2)	2.8	(−12.8–21.1)
		99^th^	−8.5	(−27.5–15.5)	38.6	(−5.90–104)
	7 days	90^th^	6.4	(−2.60–16.3)*	8.8	(−6.60–26.8)*
		95^th^	7.1	(−4.10–19.7)	5.4	(−4.20–16.1)
		97^th^	12.8	(2.90–23.8)	0.9	(−14.7–19.3)
		98^th^	9.3	(−8.20–30.1)	15.8	(−8.40–46.5)
0–14 years old	2 days	90^th^	2.8	(−8.90–15.9)	3.8	(−13.4–24.4)*
		95^th^	−0.9	(−14.1–14.2)	11.9	(−8.60–37.0)*
		97^th^	−4	(−24.2–21.5)	11.3	(−7.60–34.0)
		98^th^	0.7	(−23.2–32.0)	−0.1	(−20.1–25.0)
		99^th^	2.2	(−25.3–39.8)	−7.7	(−32.7–26.7)
	4 days	90^th^	3.9	(−9.70–19.7)	3.1	(−10.4–18.7)
		95^th^	−3	(−22.1–20.9)	18.3	(−17.6–69.9)**
		97^th^	7	(−23.5–49.8)	−1.7	(−26.4–31.4)
		98^th^	−4.9	(−35.5–40.3)	−3.8	(−36.3–45.2)
		99^th^	17.7	(−28.4–93.8)	−38.5	(−77.4–67.6)
	7 days	90^th^	2.7	(−9.40–16.5)	14.9	(−11.6–49.4)
		95^th^	2.9	(−12.3–20.9)	5.5	(−13.7–28.9)
		97^th^	0.1	(−26.6–36.6)	−0.5	(−43.3–74.7)
		98^th^	−2.5	(−27.4–30.7)	−3.1	(−42.0–62.1)
15–64 years old	2 days	90^th^	10.8	(1.30–21.3) *	−2.8	(−7.90–2.60)
		95^th^	13.1	(0.70–26.9) **	−2.3	(−8.00–3.70)
		97^th^	22.8	(4.70–43.8)	−7.8	(−15.9–1.10)
		98^th^	14.8	(−8.80–44.5)**	−5.7	(−15.5–5.20)
		99^th^	13.6	(−13.3–48.9)**	−5.7	(−20.2–11.4)
	4 days	90^th^	12.5	(1.60–24.5)	−0.8	(−11.9–11.7)*
		95^th^	12.8	(2.30–24.4)	−0.7	(−8.40–7.60)
		97^th^	26.4	(10.7–44.3)	−10.8	(−21.8–1.70)
		98^th^	14	(−12.9–49.2)	−10.1	(−25.4–8.30)
		99^th^	−3.1	(−24.1–23.7)	31.9	(−12.6–99.0)
	7 days	90^th^	11.3	(−0.30–24.2)**	−2.9	(−11.2–6.20)
		95^th^	11.8	(−1.70–27.1)**	−2.4	(−12.0–8.40)
		97^th^	14.6	(3.60–26.8)	−8	(−23.9–11.2)
		98^th^	16.5	(3.40–31.1)	−11.2	(−40.8–33.1)
> 64 years old	2 days	90^th^	19.4	(10.9–28.7)	−4.1	(−10.1–2.30)
		95^th^	21.4	(8.90–35.3)	−3.3	(−11.1–5.10)
		97^th^	37.9	(21.9–56.1)	−7.3	(−16.8–3.30
		98^th^	29.3	(4.90–59.3)	−4.2	(−15.7–9.00)
		99^th^	24.7	(−5.00–63.6)**	19.7	(−5.20–51.2)
	4 days	90^th^	22.7	(11.4–35.2)	−2	(−9.70–6.50)
		95^th^	23.4	(9.20–39.4)	−2.4	(−11.3–7.40)
		97^th^	29	(12.7–47.6)	13.3	(−2.00–31.0)
		98^th^	22.8	(−2.60–54.9)	20.7	(−0.70–46.6)
		99^th^	10.6	(−16.6–46.8)	53	(−2.80–141)
	7 days	90^th^	14.5	(0.60–30.4)**	8.2	(−3.90–21.9)
		95^th^	14.6	(−1.30–33.1)**	9.2	(−3.30–23.2)
		97^th^	24.4	(10.8–39.8)	6.1	(−17.6–36.7)
		98^th^	27.9	(11.6–46.5)	24.3	(−5.80–64.0)

***p-value < 0.001; **p-value < 0.005; *p-value < 0.10.

To date, there is no definite risk attribution for sex-related risks in relation to HW. Previous studies have shown that females have higher risks compared to males, which may be attributable to socioeconomic factors and geographical context [34,35]. On the other hand, Basu and Ostro [36] noted that sex may not play any big difference with the risk. However, similar to Bai et al. [37], we have observed that males have greater risks than females, possibly due to the nature of their work, which mostly entail working outdoors. Outdoor workers, especially those with labor intensive workloads requiring high physical activity under the sun, are vulnerable to heat stress during hot days [38]. In Thailand, construction workers reported suffering from severe heat strain due to the physically strenuous workload [39]. Heat stress, aside from the physical strain it imposes on the body, affects workers through heat exhaustion, which may lead to heat stroke and eventual death [40].

Among cause-specific risks, high effect estimates, in either main or HW, were predominantly observed among the population who died from respiratory-related conditions. High temperature may deteriorate those people suffering from chronic pulmonary diseases, thereby pre-disposing them to greater risks [11]. Michelozzi et al. [12] also note similar findings, whereby extreme temperatures may exacerbate chronic pulmonary diseases due to excess heat dissipation through circulatory adjustment. On the other hand, we have observed significant but highly uncertain estimates of protective HW effects observed in 2-day 95th temperature percentile (−9.5%; 95% CI = −31.1–18.8) and 4-day, 95th temperature percentile (−6.5%; 95% CI = −28.8–22.9). There is no clear causal pathway on how HW may be protective for respiratory-related diseases; however, in a respiratory-related condition such as asthma, an experimental study has shown that warm moist air was observed to protect the airway disruption associated with exercise [41]. Most of the HW risks in the cause-specific mortality were protective, which may be linked to an unmeasured factor such as human behavior, whereby people with pre-existing conditions would opt to stay indoors to avoid the intense heat that could affect their current physical state [42].

Early detection of the risks associated with HW are essential for both case management and early warning precautions. The utilization of the HW definitions are entirely based on the specific population of interest. For the general population, since most of the risks associated with HW days in the 2- and 4-day durations in different temperature percentiles, except for the 98th/99th temperature percentiles, were protective, issuance may deem to be of less priority, with more focus on the main temperature effects. However, using the 7-day duration at the 90th temperature percentile, statistically insignificant, but considerably elevated risks by 3.40% (−7.20–15.1), may prove to be an indication for a candidate HW warning issuance. On the other hand, HW warning issuance for the risk population would be primarily based on the 90th and/or 95th temperature percentiles, whereby risks are well pronounced among the younger (0–14 years old) and the male populations. Although some of the risks are statistically significant, caution should be exercised when interpreting the changes in the risks, since there were considerable uncertainties in the effects estimates, as observed in the wide confidence intervals. Nevertheless, we believe that the results of this study may provide relevant information needed for strategies in relation to risks associated with HW, particularly for specific risk populations. The results of this study can be of use to improve (1) child care and management strategies taking into consideration the associated risks, as well as (2) workplace policies, which would safeguard the workers’ health during HW events.

## Conclusions

This study was able to determine the extent of attributable main or HW effects among the tropical cities of the Philippines. Although there were no significant HW risks in the general population, after stratification, significant HW effects were more apparent for the younger and male populations. Relevant policies can be, therefore, drafted in relation to the risks observed in this study, which would benefit either the general or risk populations.

## Paper context

Extensive research has been done to estimate the effects of temperature on mortality, with only a few studies estimating the added heat wave effects. Tropical countries, such as the Philippines, have limited information about the effects of HW. This study estimated the risks attributable to both the main temperature effects and the HW effects. Results of this study are particularly relevant for health managers, taking into consideration the populations at risk.
